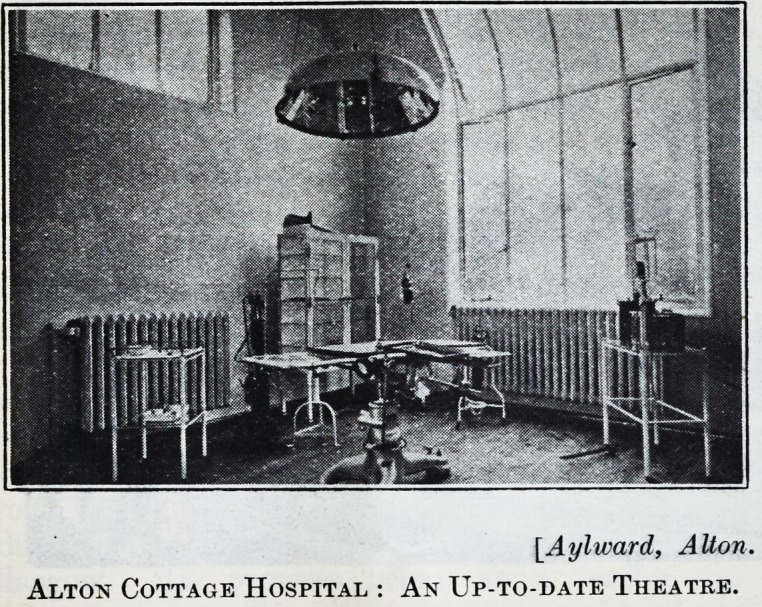# Alton Cottage Hospital Extension

**Published:** 1924-11

**Authors:** 


### ALTON COTTAGE HOSPITAL EXTENSION.

Alton's claim to medical fame has hitherto rested on the Treloar Children's Home, celebrated for its heliotherapy. The little Hampshire town may now feel justifiably proud of one of the finest of cottage hospitals, enlarged and improved by the generosity of its chairman, Mr. F. B. Summers, in memory of two of his children. Mr. Summers has provided the hospital with an electric lighting and central heating installation, new matron's and sisters' sitting-rooms, two wards each with ten beds, a most modern and convenient out-patients' department, and an X-ray room, under the control of Dr. Jaggers of Winchester, about which Röntgen himself might have been dithyrambic. The complete equipment and furnishing of these have also been part of Mr. Summers's gift.

The operating theatre, provided by public subscription, commemorates the late Mr. Basil Peel, who for eighteen years was the hospital's secretary.

[*Aylward, Alton.*

ALTON COTTAGE HOSPITAL : AN UP-TO-DATE THEATRE.

It is built and fitted with the latest devices for lighting by daylight and electricity and for sterilising, and has a roomy and convenient anæsthetising room. In the unavoidable absence of the Lord-Lieutenant of Hampshire, Major General Seely, the opening of the extension was performed by Canon Causton, who was secretary of the hospital from 1882 to 1885, and is, moreover, the son of the original founder of the hospital in 1868.

## Figures and Tables

**Figure f1:**